# Giant Gastric Bezoar Complicating Congenital Esophageal Atresia Repaired by Gastric Interposition—A Case Report

**DOI:** 10.3389/fped.2017.00098

**Published:** 2017-06-19

**Authors:** Archana Chacko, Brent I. Masters, Alan Isles

**Affiliations:** ^1^Department of Respiratory Medicine, Lady Cilento Children’s Hospital, Brisbane, QLD, Australia; ^2^The Queensland Children’s Medical Research Institute, Brisbane, QLD, Australia; ^3^The University of Queensland, Brisbane, QLD, Australia

**Keywords:** bezoars, phytobezoar, delayed gastric emptying, gastric interposition surgery, esophageal atresia

## Abstract

We describe a giant gastric phytobezoar in a child with repaired congenital esophageal atresia. At age two, a gastric interposition (pull-up) procedure was performed for severe and recurrent esophageal strictures. For 12 months post-gastric interposition, he experienced frequent respiratory illnesses requiring hospital admissions but it was not initially appreciated that these episodes were likely secondary to recurrent aspiration from a gastric bezoar with “spill-over” aspiration.

## Introduction

A bezoar is a tightly packed mass of foreign indigestible material in the gastrointestinal tract, most often in the stomach but they can occur elsewhere in the intestine. The composition of bezoars varies with different types described including phytobezoars which comprised most commonly indigestible fruits but also vegetable fibers, skin, or seeds (1).

Bezoars can develop in patients with altered gastrointestinal anatomy, motility, and even in those with otherwise normal gastrointestinal tract. Most bezoars are found in children and young females with pica, and particularly when associated with psychiatric disorders or mental retardation. By contrast, in adults, most bezoars are related to previous gastric surgery that reduces gastric motility (2, 3).

## Case History

Our patient was born with esophageal atresia without a fistula and underwent a successful primary anastomosis on day 2 of life. Beginning in his second year of life, he became symptomatic with feeding difficulties due to recurrent stricture formation at the anastomotic site. Repeated dilatations proved ineffective; therefore, a gastric interposition procedure was performed. Following gastric interposition, he began experiencing recurrent acute respiratory symptoms including wheeze and had repeated acute hospital admissions. He also experienced persistent “noisy” breathing, vomiting up to 20 times per day, and persistent halitosis. Examination at the time reportedly revealed decreased breath sounds over the right middle and right lower lobes.

**Figure 1:**
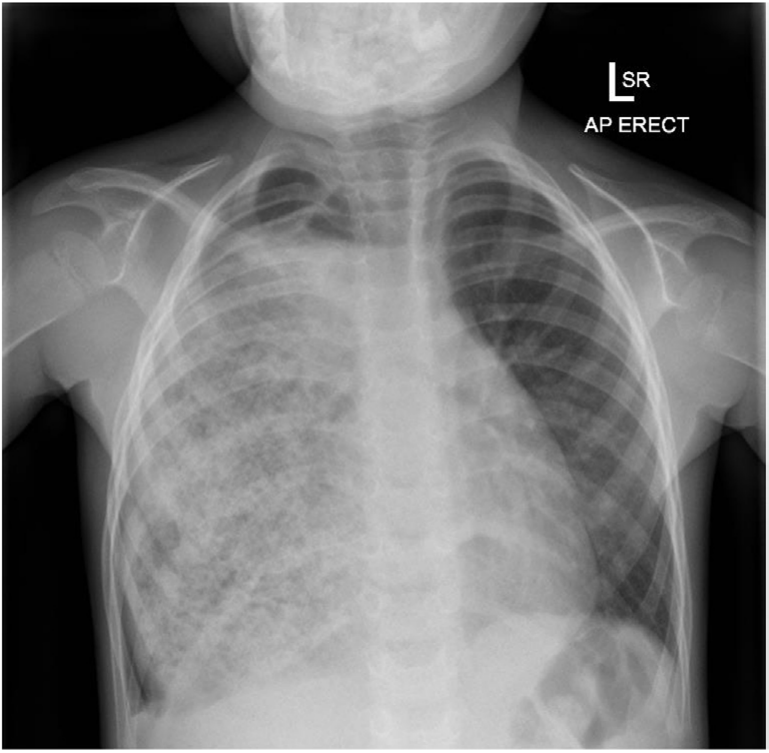
**Initial CXR after referral to our services**. Right hemithorax almost totally opacified with an unusual patterned appearance to the opacity.

**Figure 2:**
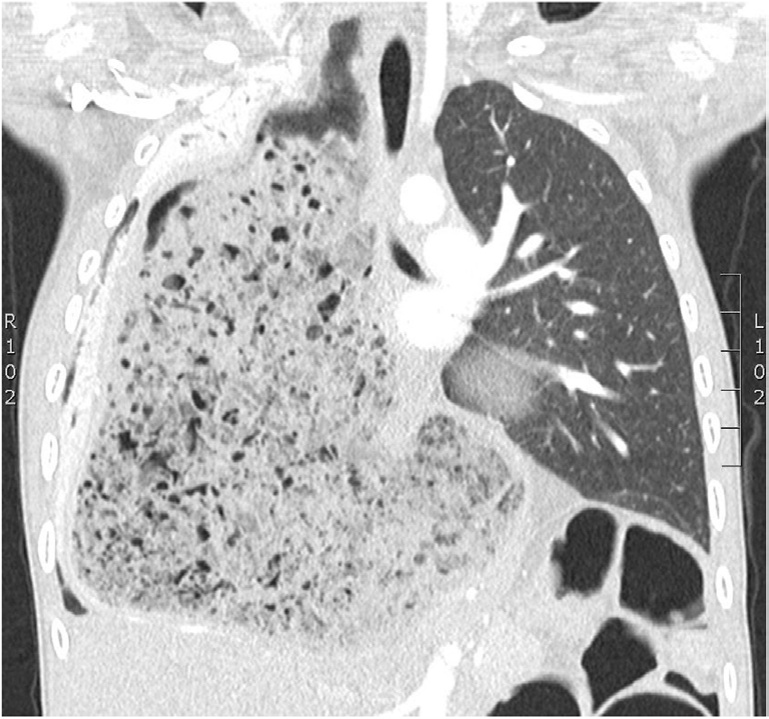
**Coronal section of chest CT scan**. Gross distension of the stomach and a giant bezoar containing gas and food debris causing unusual patterning in the right hemithorax.

**Figure 3:**
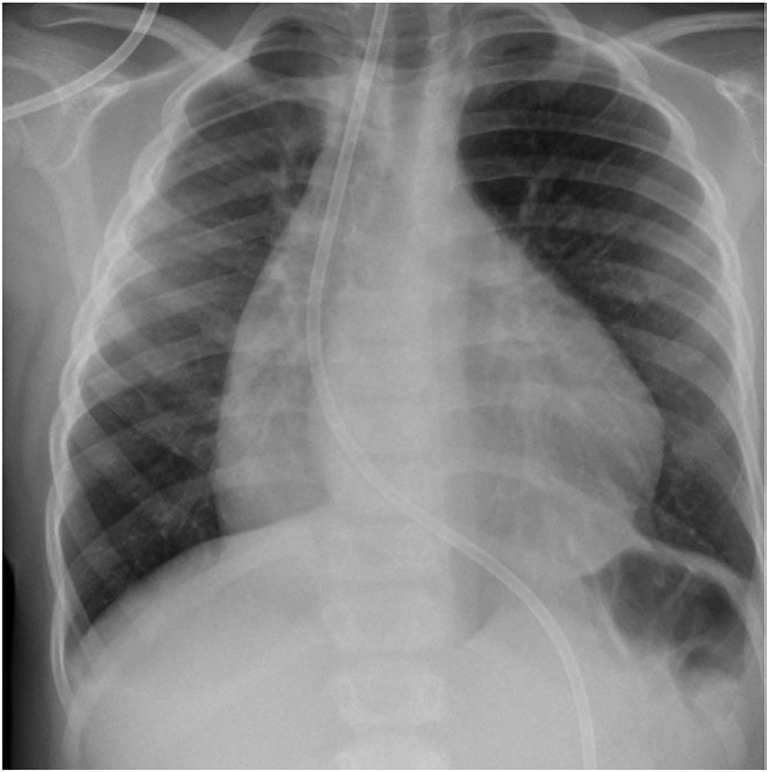
**Most recent CXR post-surgery**. The neo-esophagus is apparent at the right mediastinal border.

At presentation to our service, his chest radiograph and computed tomography were significantly abnormal with gross distension of the stomach and a giant bezoar containing gas and food debris which had caused the unusual patterning seen on his chest radiographs (Figures 1 and 2). The cause was attributed to gastric dysmotility complicating his previous gastric surgery resulting in bezoar formation with pulmonary aspiration. Bronchoscopy revealed bronchomalacia of the right main bronchus, but more importantly solid material was seen extruding from his esophageal inlet into the pyriform fossa. Gastroscopy (flexible and rigid) was unsuccessful in removing the bulk of the material. Repeated drinks of Coca Cola^®^ (250–500 ml daily) lead to dissolution of the bezoar, and ultimately successful gastroscopic removal of the bezoar. Of note on the fourth postoperative day, the patient had a significant hematemesis requiring fluid resuscitation. This was a self-limiting event and the previous endoscopic instrumentation and possible concurrent gastric ulceration related to the bezoar was suspected to be the cause. Later surgery was performed to remove a fold of redundant gastric mucosa obscuring the pylorus which was above the diaphragm and part of the stomach was reduced into the peritoneal cavity. His most recent chest radiograph has markedly improved (see Figure 3).

## Discussion

Bezoars may not cause any symptoms for years and are usually incidentally discovered. If they do the onset can be insidious. Symptoms can include abdominal pain, nausea, vomiting, early satiety, anorexia, and weight loss. Uncommon presentations include gastrointestinal bleeding due to concurrent gastric ulcers and gastric outlet obstruction. Physical examination may include an abdominal bezoar mass and halitosis; however, it is most often unremarkable (4). Our patient is unusual because of the intra-thoracic site, young age, and gastric outlet obstruction all occurring together. His propensity to eat whole prawn including the shell is also very unusual behavior for a 4-year-old child and was likened to pica.

Investigations such as abdominal radiograph, abdominal ultrasound, or CT scan may show a mass representing the bezoar. In this case, the chest X-ray provided the clue to a dilated stomach but the bronchoscopic visualization of food extending from the esophageal inlet and then upper gastrointestinal endoscopy confirmed the diagnosis (5, 6).

Bezoar management involves lavage or dissolution and fragmentation of gastric materials, and/then retrieval of as much material as possible. This usually involves forceps and suction removal of the various materials. Many substances have been utilized to effect dissolution of the gastric materials including Coca-Cola^®^ (The Coca-Cola Company) and as in our case it was successful (1, 7–9). Failing this, surgical removal has been used (10).

While the giant bezoar in this case is of interest, there are other important messages to be drawn from this case. A gastric interposition procedure may impair gastric motility. In gastric interposition, the stomach is mobilized through an epigastric incision and proximal esophagus through a neck incision. The esophageal stump is excised and pyloroplasty is performed. A trans-hiatal posterior mediastinal tunnel is created. The gastro-esophageal anastomosis is performed in the neck using the apex of the stomach (11).

In any child who has had surgical repair of esophageal atresia either by primary anastomosis or, as in this case, by a secondary gastric interposition procedure, who experiences recurrent or persistent respiratory symptoms, aspiration into the airway should be central in the differential diagnosis. With hindsight, this boy’s chest radiograph was grossly abnormal some 12 months before his referral. The significance of the radiographic changes and the likelihood of recurrent aspiration as the cause of his repeated acute respiratory events were not appreciated with these presentations misdiagnosed as pneumonia or asthma exacerbations. While an intra-thoracic bezoar is uncommon, the recurrent acute respiratory symptoms associated with frequent daily vomiting were strongly indicative of recurrent pulmonary aspiration and indications for further investigation.

## Concluding Remarks

Children who have had surgical repair of esophageal atresia are at later risk of pulmonary aspiration through stricture formation at the site of the primary anastomosis. In this case, gastric interposition surgery performed because of recurrent stricture formation was subsequently complicated by recurrent vomiting, episodes of acute respiratory distress due to pulmonary aspiration. These were all clues to disordered gastric function and emptying. While gastric bezoar formation would not, necessarily, have been “at the top of mind” as a diagnosis, the chest radiograph prior to referral was strongly suggestive of this diagnosis.

## Informed Consent

Parental consent given to present case.

## Author Contributions

AC, BM, and AI contributed to the writing and editing of the case report.

## Conflict of Interest Statement

The authors declare that the research was conducted in the absence of any commercial or financial relationships that could be construed as a potential conflict of interest.
